# Oxidative Stress and NRF2-Mediated Redox Regulation in Incomplete Systemic Lupus Erythematosus and Systemic Lupus Erythematosus

**DOI:** 10.3390/antiox15080995

**Published:** 2026-08-11

**Authors:** Lu Liu, Svenja Henning, Harry van Goor, Hendrika Bootsma, Berber Doornbos-van der Meer, Johanna Westra, Karina de Leeuw

**Affiliations:** 1 Department of Rheumatology and Clinical Immunology, University Medical Centre Groningen, University of Groningen, Hanzeplein 1, 9713 GZ Groningen, The Netherlandss.henning@umcg.nl (S.H.); h.bootsma@umcg.nl (H.B.); b.doornbos-van.der.meer@umcg.nl (B.D.-v.d.M.);; 2 Department of Pathology and Medical Biology, University Medical Centre Groningen, University of Groningen, Hanzeplein 1, 9713 GZ Groningen, The Netherlands; h.van.goor@umcg.nl

**Keywords:** oxidative stress, systemic lupus erythematosus, NF-E2-related factor 2 (Nrf2), serum free thiols, antioxidants

## Abstract

Oxidative stress plays an important role in systemic lupus erythematosus (SLE). To elucidate whether it is already present in early phases, we investigate oxidative stress-related genes in incomplete SLE (iSLE) and quiescent SLE (qSLE, inactive disease), as well as the effect of nuclear factor erythroid-derived 2-like 2 (NRF2) activators on NRF2-related genes in peripheral blood mononuclear cells (PBMCs) and HaCaT keratinocytes. In total, 28 qSLE patients, 29 iSLE patients and 21 age- and sex-matched healthy controls (HCs) were included. Serum free thiols, reactive oxygen species (ROS) levels and NRF2-related antioxidant gene expression were measured. Furthermore, PBMCs and HaCaT keratinocytes were treated with the NRF2 activators sulforaphane (SFN) and dimethyl fumarate (DMF) in vitro to assess expression of antioxidant genes. Finally, NRF2 and Heme oxygenase-1 (HMOX1) proteins in non-sun-exposed skin sections were assessed. Thiols were significantly lower in qSLE patients compared to HCs. In whole blood, Kelch-like ECH-associating protein 1 (KEAP1) and catalase (CAT) mRNA levels were significantly reduced in iSLE and qSLE. Treatment with SFN or DMF in PBMCs upregulated HMOX1 and NAD(P)H quinone dehydrogenase-1 (NQO1) mRNA expression and downregulated CAT expression. In HaCaT cells, mRNA expression of HMOX1, NQO1 and thioredoxin was upregulated. There were no differences in protein expression of NRF2 and HMOX1 in skin tissues. In conclusion, in qSLE patients, oxidative stress is elevated, while antioxidant capacity is decreased. A similar trend, although not significant, is seen in iSLE patients, which indicates that redox imbalances are already present in early phases, but not as obvious as in established SLE. NRF2 activators upregulate antioxidant gene expression in PBMCs and HaCaT cells, highlighting their potential role to modulate oxidative stress pathways in SLE.

## 1. Introduction

Systemic lupus erythematosus is an autoimmune disease involving multiple organs with heterogeneous clinical manifestations, characterized by the presence of auto-antibodies, immune complex deposition and chronic inflammation [1]. The prevalence of SLE worldwide is estimated at 43.7 cases (15.9 to 108.9) per 100 000 persons, or 3.4 million people. It predominantly affects women, with a ratio of about 9:1 [2]. The diagnosis of SLE is based on clinical and laboratory observations, and often classification criteria are used, of which the EULAR/ACR-2019 criteria are the newest [3]. Heterogeneous features and overlapping symptoms with other diseases are main challenges for early identification of SLE, resulting in delayed diagnosis [4]. In early stages of the disease, some patients present with SLE-related clinical symptoms and immunologic abnormalities that are not sufficient to meet the classification criteria. These patients are described as having incomplete SLE (iSLE), of whom up to 57% will finally progress to SLE [4].

Reactive oxygen species (ROS)/reactive nitrogen species (RNS) are partly generated by insufficient reduction of oxygen/nitrogen during redox processes. In SLE, numerous studies have shown that increased ROS/RNS levels seem to be essential for activating pathways which are implicated in the pathogenesis of this disease [5,6]. Excessive production of ROS/RNS is related to decreased antioxidant capacity and thus a reduced ability to neutralize oxidative stress, leading to more oxidative damage, disturbed redox signaling and ensuing inflammation in SLE [7]. Free thiols, organosulfur compounds containing a sulfhydryl (R-SH) moiety, are a reliable reflection of the overall in vivo reduction–oxidation (redox) status because they capture the balance between total oxidant burden and antioxidant capacity [8,9,10]. Of note, our previous study showed that low free thiol levels were present in patients with active lupus nephritis (LN), which were inversely associated with increased disease activity, so low antioxidant capacity is related to high disease activity [8].

Antioxidant mechanisms are regulated through the transcription factor NF-E2-related factor-2 (NRF2), a central regulator of cellular antioxidant responses, anti-inflammation and detoxification. Accumulating reports support an important role for the deregulation of NRF2 in SLE [11,12]. NRF2 is an unstable, short-lived protein under stable conditions. Abundance of NRF2 is regulated through binding to Kelch-like ECH-associating protein 1 (KEAP1) in the cytosol. KEAP1 is an E3 ubiquitin ligase substrate adaptor that targets NRF2 for ubiquitination and proteasomal degradation [13]. Upon oxidative stress conditions, KEAP1 serves as a sensor for oxidants and endogenous and exogenous electrophiles, due to the presence of highly reactive cysteine residues, and undergoes chemical modification. KEAP1 no longer targets NRF2 for degradation, leading to accumulation of newly synthesized NRF2, and translocation to the nucleus, a process induced, for example, by sulforaphane (SFN) and dimethyl fumarate (DMF), where it binds to antioxidant response element and promotes transcription of antioxidant genes such as heme oxygenase (HMOX1), catalase (CAT), thioredoxin (TXN), and NAD(P)H quinone oxidoreductase-1 (NQO1), thereby enhancing cellular protection [14,15].

Many compounds are known to exert antioxidant properties; among these, sulforaphane (SFN) and dimethyl fumarates (DMF) are two potent activators of NRF2 [16,17]. SFN is enriched in cruciferous vegetables such as broccoli sprouts and has been shown to induce cellular defense [18]. DMF is a fumaric acid ester that can oxidize sulfhydryl (-SH) moieties of KEAP1. Recently, numerous studies have demonstrated that DMF has anti-inflammatory and cytoprotective properties, and it has been approved in clinical trials for treatment of psoriasis and remitting–relapsing multiple sclerosis [17,19,20]. Furthermore, studies have shown that NRF2-inducing agents have beneficial effects in SLE mouse models [21]. However, the role of NRF2 activators in SLE patients needs to be further explored. Also, little is known about the oxidative status of iSLE patients, who present with features of SLE but do not fulfill all the criteria, which can be seen as one of the earliest stages in the disease progression.

Therefore, the aim of this study was to investigate levels of serum free thiols, reflecting systemic oxidative stress, as well as the expression of NRF2 and its related downstream target genes in patients with quiescent SLE, patients with iSLE and healthy controls (HCs), both in peripheral blood and in skin tissues. Moreover, to investigate the effect of SFN and DMF on NRF2 activation, peripheral blood mononuclear cells (PBMCs) from patients with qSLE, patients with iSLE and HCs were stimulated, as well as HaCaT keratinocytes, a human keratinocyte cell line, which was used as a skin model, since many iSLE patients present with skin lesions. Investigating the role of oxidative stress and NRF2-mediated redox regulation in iSLE patients could provide novel insights into the role of oxidative stress and pharmaceutic NRF2 activation in the early stages of the progression of SLE.

## 2. Material and Methods

### 2.1. Study Population

Patients with qSLE, patients with iSLE and HCs were included, with cross-sectional data from the iSLE cohort study at the University Medical Centre Groningen [22]. The included iSLE patients had ANA titers of ≥1:80, disease duration of <5 years at enrollment and at least one of the Systemic Lupus International Collaborating Clinics (SLICC) criteria [23,24], but not enough criteria to classify as SLE. Some iSLE patients were treated with hydroxychloroquine (HCQ), but no other immunosuppressants. All qSLE patients met the ACR or SLICC criteria [23,24], and had inactive or quiescent disease, defined as an SLE disease activity index (SLEDAI) < 5. Demographic and disease-related information of participants was assessed and recorded by a physician; disease activity was assessed with the SLEDAI [25]. HCs were included if they had no history of autoimmune disease and no infection at the time of inclusion. PBMCs were isolated by Ficoll–Paque density gradient centrifugation and stored in liquid nitrogen. Serum and plasma samples were stored at −20 °C until processing. For mRNA analysis, blood was drawn into Paxgene tubes and stored at −80 °C. The study was conducted at the University Medical Centre Groningen in line with the declaration of Helsinki, and was approved by the medical ethics committee (METc2015/313). All participants gave written informed consent.

### 2.2. Serum Free Thiols

The levels of thiols (µmol/L) were measured for 21 HCs, 23 iSLE patients and 27 SLE patients using the Measure-iT™ thiol assay kit (Thermo Fisher Scientific, Waltham, MA, USA, #M30550) according to the manufacturer’s protocol. Briefly, a series of thiol quantitation standard was prepared; 100 µL of quantitation reagent was loaded to each microplate well, followed by adding 10 µL of thiol quantitation standard or adding 1–10 µL of each patient and control serum samples. The volumes of all reactions were equalized with dilution buffer if necessary. Colorimetric measurement was performed using a Sunrise microplate reader (Tecan Trading AG, Männedorf, Switzerland) with a reference filter at 494/517 nm.

### 2.3. Gene Expression of Anti-Oxidative Stress Markers by RT-PCR in Whole Blood

Total RNA was extracted from whole blood using the Paxgene Blood RNA Kit (Thermo Fisher Scientific, Waltham, MA, USA, #762164) according to the manufacturer’s instructions. Isolated RNA was reverse-transcribed to cDNA and subsequently quantitatively analyzed using an Applied Biosystems™ QuantStudio™ 6 Flex Real-Time PCR System. The following Taqman primers and probes were used: NFE2L2/NRF2 [Hs00975960_m1], KEAP1 [Hs00202227_m1], HMOX1 [Hs01110250_m1], CAT [Hs00156308_m1], TXN [Hs00828652_m1], NQO1 [Hs00168547_m1], and GAPDH [Hs99999905_m1] (Applied Biosystems, Warrington, UK). Relative expression (RE) was calculated based on the cycle threshold (Ct) value related to expression of the housekeeping gene GAPDH as follows: RE = 2^−(Ct Test gene−Ct GAPDH)^. Fold induction was calculated as 2^−[∆∆Ct (∆Ct test gene stim−∆Ct test gene unstim)]^. Data were analyzed using Quant Studio Real-Time PCR software v1.3 (Applied Biosystems, Singapore).

### 2.4. Activation of NRF2 Pathway with SFN and DMF in PBMCs

Frozen PBMCs were thawed in RPMI with fetal calf serum (FCS). Immediately after thawing PBMC viability was checked (>95% viable) and suspensions at a concentration of 1 × 10^6^ cells/mL in RPMI/1% FCS were made, after which 500 µL cell suspension was added to tubes. Next, 500 µL of 40 µM SFN (final concentration 20 µM) or 500 µL 80 µM DMF (final concentration 40 µM) was added to the tubes, which were then incubated for 6 h at 37 °C and 5% CO_2_. Optimal concentrations of SFN and DMF were previously established. After centrifugation, supernatants were removed and cells were lysed with 1 mL TRIzol (Thermo Fisher Scientific, Waltham, MA, USA, # 15596026) and stored at −80 °C until analysis. mRNA was isolated according to standard protocols, and cDNA transcription and RT-PCR were performed as described above.

### 2.5. Measurement of ROS Levels in PBMCs

Intracellular ROS levels were detected by using a DCFDA/H2DCFDA-cellular ROS assay kit (Abcam, Cambridge, UK, #ab113851) according to the manufacturer’s protocol. DCFDA (Dichlorodihydrofluorescein Diacetate) is a cell-permeable dye that is oxidized to fluorescent DCF by ROS. Briefly, 2 × 10^5^ thawed PBMCs were put into tubes. Cells pre-treated with TBHP (tert-Butyl hydroperoxide) solution were used as an intracellular ROS-positive control. Cells were incubated with 200 µL of 20 µM DCFDA solution at 37 °C in the dark for 30 min. Then, cells were washed and centrifuged. After resuspension and addition of buffer with 10% FBS (fetal bovine serum), cells were plated in 96-well plates (100 µL/well) and incubated at 37 °C in the dark for 3 h. Fluorescence was read in a fluorescence plate reader at Ex/Em = 485/535 nm.

### 2.6. Activation of the NRF2 Pathway with SFN and DMF in HaCaT Keratinocytes

As a model for NRF2 activation in skin, HaCaT cells, a spontaneously transformed aneuploid immortal keratinocyte cell line from adult human skin, were used. HaCaT cells were grown in Keratinocyte-SFM basal medium (Gibco, #17-005-042, Thermo Fisher Scientific, Waltham, MA, USA) enriched with 50 μg/mL bovine pituitary extract (BPE), 5 ng/mL human recombinant epidermal growth factor (EGF) and 60 μg/mL gentamycin and passaged at a confluence of 80%. For experiments, 4 × 10^5^ cells per well were plated in 24-well plates and left to adhere for 24 h. Then cells were treated with 20 µM H_2_O_2_, 20 µM SFN or 40 µM DMF for 6 or 24 h. After stimulation, RNA from HaCaT was extracted from cell lysates using the RNeasy Mini Kit^®^ (Qiagen, Venlo, The Netherlands) according to the manufacturer’s instructions, and cDNA transcription and RT-PCR were performed as described above.

### 2.7. Immunohistochemistry of Skin Sections

We cut 4 µm thick sections from paraffin-embedded skin tissues from a subset of HCs, as well as iSLE and qSLE patients. Biopsies were from unaffected and non-sun-exposed skin from the buttock [22]. After deparaffinization and antigen retrieval, the tissue sections were stained using antibodies against NRF2 (Abcam, Cambridge, UK, #ab62532) and Heme oxygenase (HMOX, Abcam, Cambridge, UK, #ab68447), followed by incubation with goat anti-rabbit Ig/HRP (DAKO, Glostrup, Danmark #0448) and development with Dako Liquid DAB+ Substrate Chromogen System and counterstaining with hematoxylin. All slides were coded and evaluated in a blinded fashion. Staining was graded from 0 to 4 semi-quantitatively as follows: grade 0, no expression; grade 1, weak expression; grade 2, moderate expression; grade 3, intermediate expression; grade 4, strong expression. Scoring was performed for epidermis, endothelium, infiltrates, fibroblasts and adnexa sections. Statistical analysis could not be performed due to the relative low number of these samples.

### 2.8. Statistical Analysis

Data are presented as mean ± standard deviation (SD) for normally distributed and median with interquartile range (IQR: p25–p75) for non-normally distributed continuous variables and number of patients (percentage) for categorical variables. Demographic, clinical, and laboratory characteristics and medication use were compared between patients with qSLE and iSLE using Independent Samples *t*-test, or Mann–Whitney-U test for non-normal-distribution data and Chi-Square test as appropriate. Differences between three groups in Nrf2-related downstream signaling pathways were analyzed using Kruskal–Wallis test. Spearman correlation coefficients were used to analyze the correlations. Data analysis was performed using SPSS version 28 and graphs were made with GraphPad Prism 9.

## 3. Results

### 3.1. Patient Characteristics

Characteristics of 28 SLE patients, 29 iSLE patients and 21 HCs for the measurements of NRF2-related gene expression in whole blood and serum free thiols are shown in Table 1 (total group). Characteristics of 15 SLE patients, 20 iSLE patients and 10 HCs (subgroup), for whom ROS levels and activation experiments with PBMCs were measured, are shown in Table 2. Most patients were female, the median age was approximately 40 years, and all had low disease activity without active nephritis or active skin lesions. Of the 28 SLE patients, nine patients had nephritis in the past. Overall, patients with qSLE had longer time since diagnosis compared to iSLE patients (*p* = 0.003). There were more smokers among iSLE (38%) than qSLE patients (14%), and anti-dsDNA and serum creatinine levels were significantly higher in qSLE compared to iSLE patients. Hydroxychloroquine (HCQ) was used by 93% of qSLE patients and 31% of iSLE patients. Prednisolone and mycophenolate mofetil were used by 25% of qSLE patients, but not by iSLE patients.

### 3.2. Levels of Serum Free Thiols

**Figure 1:**
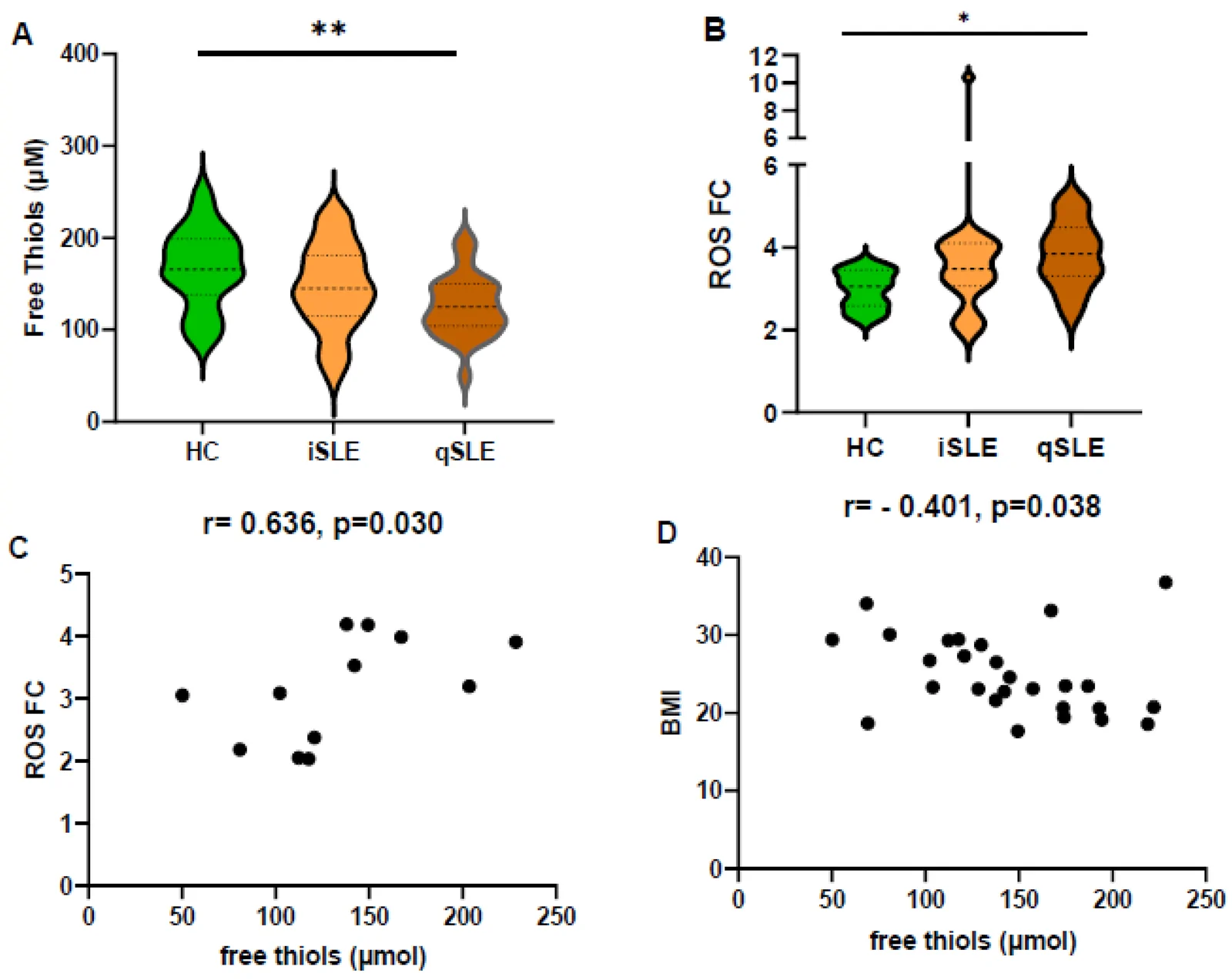
**Levels of free thiols in serum and ROS in PBMCs of healthy controls, iSLE patients and qSLE patients, and correlations.** Violin plots of levels of serum free thiols in HCs, iSLE patients and qSLE patients (**A**), and of ROS levels in unstimulated PBMCs of HCs, iSLE patients and qSLE patients (**B**). Correlation between serum free thiol levels and ROS levels in PBMCs of iSLE patients (**C**); correlation between serum free thiols and BMI of iSLE patients (**D**). Fold change (FC) in ROS levels was calculated from fluorescence of stained cells compared to unstained cells. HC: healthy controls; iSLE: incomplete systemic lupus erythematosus, qSLE: quiescent SLE; * *p* < 0.05, ** *p* < 0.01.

Overall, the levels of serum free thiols were different between qSLE patients, iSLE patients and HCs when comparing all groups (Kruskal–Wallis test, *p* = 0.007). Comparisons between separate groups showed that free thiol levels were significantly lower in qSLE patients compared to HCs (*p* = 0.001), while there were no significant differences between neither HCs and iSLE nor iSLE and qSLE (*p* = 0.126, *p* = 0.083 respectively), as shown in Figure 1A.

### 3.3. Intracellular ROS Levels in Unstimulated PBMCs and Correlations

Comparisons between groups showed that ROS levels in PBMCs were higher in qSLE patients compared to HCs (*p* = 0.011), but there was no significant difference between iSLE patients and HCs, as shown in Figure 1B. In 12 iSLE patients, both serum free thiols in whole blood and ROS levels in PBMCs were measured and a significant correlation was found (*p* = 0.030), as shown in Figure 1C; these correlations were not found in qSLE and HC, possibly because of low numbers of samples in which both thiols an ROS levels were available. A significant inverse correlation between free thiols and BMI was seen in iSLE patients (*p* = 0.038) (Figure 1D). There were no differences between iSLE patients with or without HCQ use regarding free thiols, ROS levels, and NRF2 mRNA expression. Also, no differences in levels of these markers were seen for qSLE patients on steroids or MMF. No other correlation between thiol or ROS levels with clinical or biochemical measures were found, probably due to small numbers.

### 3.4. Expression of NRF2 mRNA and Its Related Downstream Target Genes in Whole Blood

**Figure 2:**
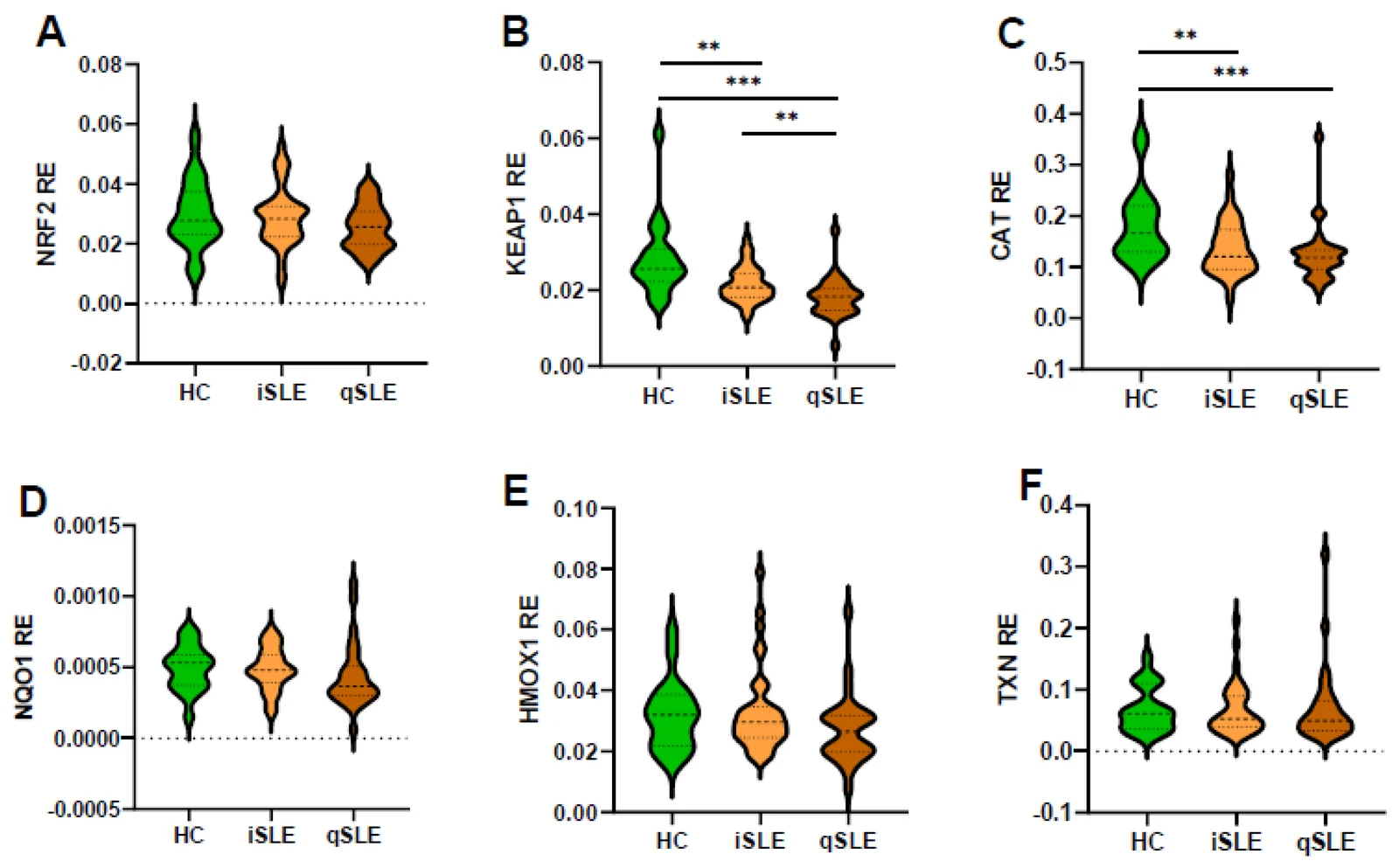
**Violin plots of NRF2mRNA expression and downstream targets in whole blood of healthy controls, iSLE patients and qSLE patients.** RT-qPCR was used to examine mRNA expression of NRF2 (**A**), KEAP1 (**B**), CAT (**C**), NQO1 (**D**), HMOX1 (**E**), and TXN (**F**) of HC, iSLE patients and SLE patients. Violin plots are presented; horizontal lines in each box plot represent median with interquartile range (IQR). ** *p* < 0.01, *** *p* < 0.001, RE: relative expression; HCs: healthy controls; iSLE: incomplete systemic lupus erythematosus; qSLE: quiescent SLE; NRF2: Nuclear factor erythroid 2-related factor 2; KEAP1: Kelch like-ECH-associated protein 1; CAT: catalase; HMOX1: heme oxygenase 1; NQO1: NAD(P)H quinone dehydrogenase 1; TXN: Thioredoxin.

Expression of mRNA of NRF2, KEAP1 and downstream antioxidant genes was measured in whole blood samples of HCs, iSLE patients and qSLE patients. NRF2 mRNA expression did not differ between groups (Figure 2A); however, given that NRF2 activity is primarily regulated at the protein stability level through KEAP1-mediated mechanisms, unchanged NRF2 transcript levels do not exclude alterations in NRF2 pathway activity. Comparisons between groups showed that expression of KEAP1 was significantly reduced in iSLE and qSLE patients compared to HCs, and in qSLE compared to iSLE patients (*p* = 0.007, *p* < 0.001, *p* = 0.004, respectively) (Figure 2B). Expression of CAT was significantly reduced in both iSLE and SLE patients compared to HCs (*p* = 0.002 and *p* = 0.001) (Figure 2C). Furthermore, there were no significant differences between the groups regarding other antioxidant gene expression, as shown in Figure 2D–F.

In patients with iSLE, NRF2 expression was positively associated with CAT and TXN expression (rho = 0.56, *p* = 0.002; rho = 0.50, *p* = 0.007, respectively), and KEAP1 was positively associated with HMOX1 and NQO1 expression (rho = 0.42, *p* = 0.025; rho = 0.39, *p* = 0.038, respectively), as shown in Supplementary Figure S1A. In patients with qSLE, NRF2 expression was positively associated with KEAP1, TXN, HMOX1 and NQO1 expression (rho = 0.64, *p* < 0.001; rho = 0.50, *p* = 0.007; rho = 0.57, *p* = 0.002; rho = 0.42, *p* = 0.027, respectively). KEAP1 expression was positively associated with TXN and HMOX1 expression (rho = 0.37, *p* = 0.052; rho = 0.56, *p* = 0.002, respectively); see Supplementary Figure S1B.

### 3.5. Activation Studies

#### 3.5.1. mRNA Expression of Antioxidant Genes in PBMCs Treated with NRF2 Activators

**Figure 3:**
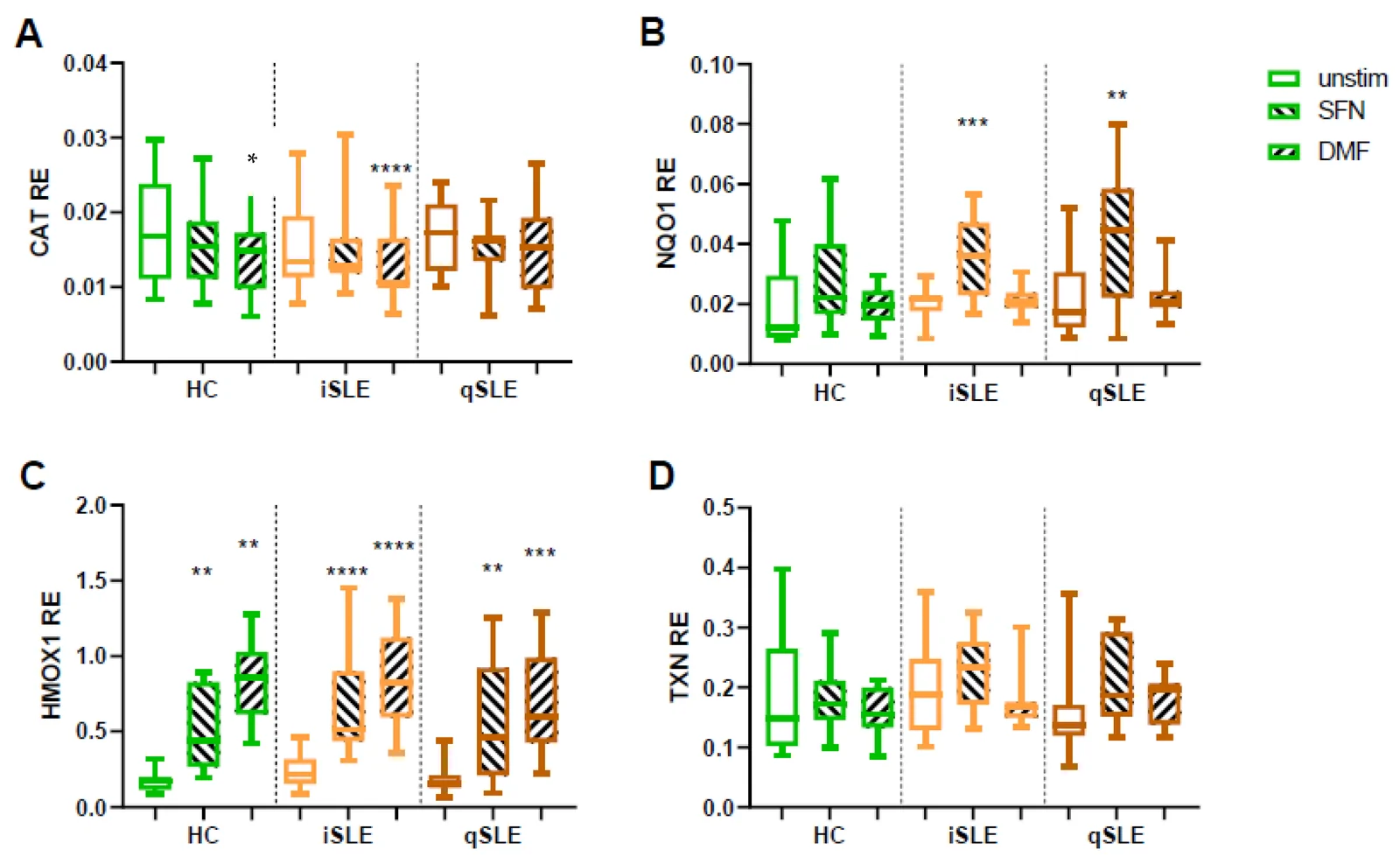
**Box plots of mRNA relative expression of antioxidant genes in unstimulated and stimulated PBMCs of HCs, iSLE patients and qSLE patients.** mRNA relative expression of CAT (**A**), NQO1 (**B**), HMOX1 (**C**), TXN (**D**), in unstimulated and stimulated PBMCs of HCs, iSLE and qSLE. Box plots are presented; horizontal lines in each box plot represent median with interquartile range (IQR) with minimal and maximal values as whiskers. All mRNA expression assessments were performed in duplicate. Box plots in green represent HCs, those in orange represent iSLE, and those in brown represent qSLE; * *p* < 0.05, ** *p* < 0.01, *** *p* < 0.001, **** *p* < 0.0001, compared to their respective unstimulated control by using Wilcoxon matched-pairs test. RE: relative expression; HCs: healthy controls; iSLE: incomplete systemic lupus; SFN: sulforaphane; DMF: dimethyl fumarate; CAT: catalase; HMOX1: heme oxygenase 1; NQO1: NAD(P)H quinone dehydrogenase 1; TXN: thioredoxin.

PBMCs were treated with 20 µM SFN or 40 µM DMF for 6 h in vitro. Next, mRNA expression of NRF2 downstream genes was measured with RT-PCR. CAT expression was significantly downregulated in PBMCs from HCs and iSLE patients treated with DMF (*p* = 0.014 and *p* < 0.001) (Figure 3A). NQO1 expression was significantly upregulated in PBMCs from patients with iSLE and qSLE treated with SFN (*p* = 0.014, *p* = 0.005, respectively) (Figure 3B). Of interest, HMOX1 expression was strongly upregulated in PBMCs from qSLE patients, iSLE patients and HCs treated with SFN (*p* = 0.008, *p* < 0.001 and *p* = 0.003, respectively) and DMF (*p* = 0.004, *p* < 0.001 and *p* < 0.001, respectively) (Figure 3C). No significant differences were found for TXN expression (Figure 3D).

#### 3.5.2. Expression of Antioxidant Genes in HaCaT Keratinocytes Treated with NRF2 Activators

**Figure 4:**
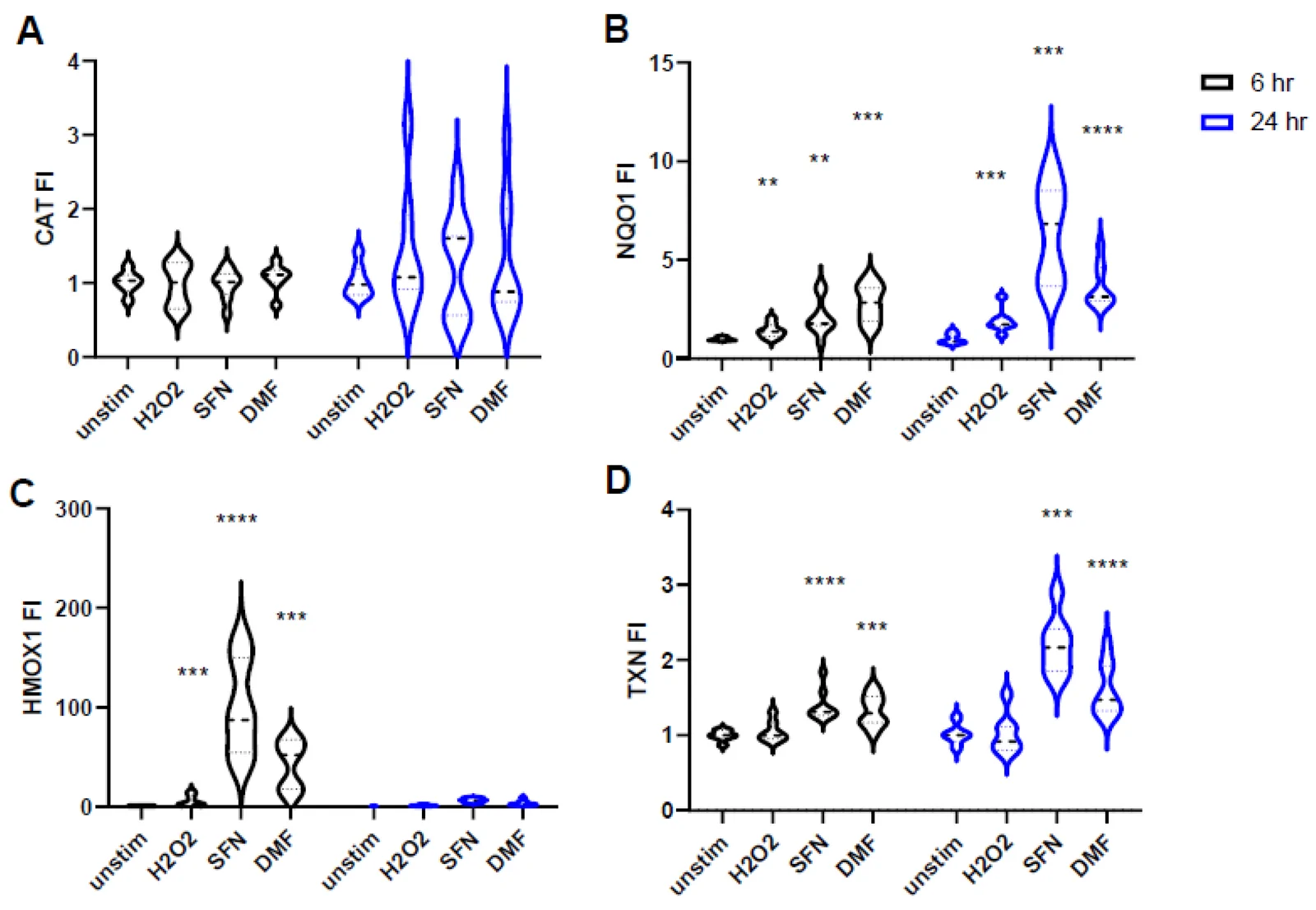
**Violin plots of mRNA expression of NRF2 downstream target in HaCaT cells treated with H_2_O_2_, SFN or DMF.** mRNA fold induction of CAT (**A**), NQO1 (**B**), HMOX1 (**C**), TXN (**D**) in HaCaT cells treated with H_2_O_2_, SFN or DMF for either 6 h or 24 h as marked in figure. Box plots with all individual values are presented; horizontal lines in each box plot represent median with interquartile range (IQR) with minimal and maximal values as whiskers. Individual values are shown as dots. ** *p* < 0.01, *** *p* < 0.001, **** *p* < 0.0001, compared to their respective unstimulated control in subgroups by using Mann–Whitney test. Subgroups are separated by dashed lines. FI: Fold induction;; SFN: sulforaphane; DMF: dimethyl fumarates; Nrf2: Nuclear factor erythroid 2-related factor 2; CAT: catalase; HMOX1: heme oxygenase 1; NQO1: NAD(P)H quinone dehydrogenase 1; TXN: Thioredoxin.

We next determined the impact of 20 µM H_2_O_2_, to mimic oxidative stress, and of 20 µM SFN and 40 µM DMF activation on expression of antioxidant genes in HaCaT keratinocytes. After 6 h of treatment, HMOX1, NQO1 and TXN expressions were significantly upregulated compared to unstimulated samples, with the exception of TXN expression in H_2_O_2_-treated cells, as shown in Figure 4A–D. After 24 h of treatment, only NQO1 and TXN expressions remained significantly upregulated, except for TXN expression in H_2_O_2_-treated samples; see Figure 4B,D. CAT expression was not upregulated after treatment in HaCaT cells.

#### 3.5.3. Skin Biopsies

**Figure 5:**
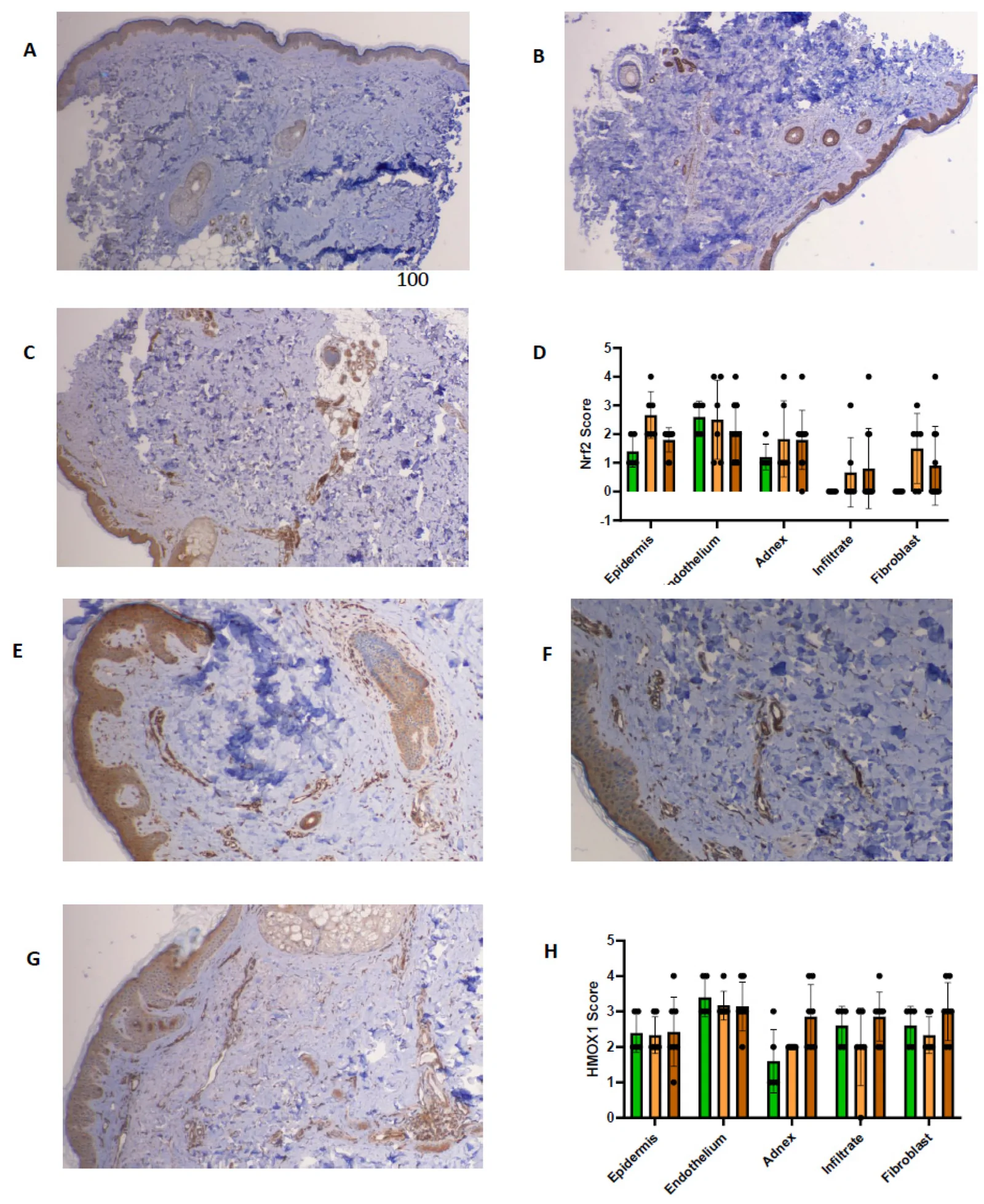
**Immunohistochemistry staining of NRF2 and HMXO1 in a representative non-sun-exposed skin sections from HCs, iSLE and SLE**. NRF2 staining of skin tissues in HCs (**A**), iSLE (**B**) and SLE (**C**); HMOX1 staining of skin tissues in HCs (**E**), iSLE (**F**) and SLE (**G**); scoring of Nrf2 (**D**) and HMOX1 (**H**) on epidermis, endothelium, adnexa, infiltrate and fibroblast, where values are presented as mean ± SEM, *n* = 1–10/group. In histograms, green bars represent HCs, orange bars represent iSLE and brown bars represent qSLE. NRF2: Nuclear factor erythroid 2-related factor 2; HMOX1: heme oxygenase 1.

Expression of NRF2 and HMOX1 protein was detected in non-lesional, non-sun-exposed skin sections of HCs and patients, as illustrated in Figure 5A–C,E–G. Semi-quantitative evaluation of these proteins showed no differences between the groups in epidermis, endothelium, adnexa, infiltrate and fibroblast (Figure 5D,H).

## 4. Discussion

In this cross-sectional study, we found significantly increased oxidative stress, as shown by reduced serum free thiol levels and increased ROS levels, in PBMCs in patients with qSLE. Although levels of thiols and ROS were inversely correlated in iSLE patients, the levels were not significantly different compared to qSLE and HCs. Expression of KEAP1 and CAT mRNA was significantly reduced in qSLE and iSLE patients compared to HCs. Treatment of PBMCs with NRF2 activators SFN and DMF strongly upregulated HMOX1 gene expression in all groups. In HaCaT keratinocytes, SFN and DMF activators showed the ability to upregulate NRF2 downstream genes, such as HMOX1, NQO1 and TXN. Overall, these results highlight the potential of NRF2 activation by agonists like SFN and DMF as a novel adjunctive therapy for SLE, potentially already in earlier stages of the pathogenesis.

Serum free thiols can reliably reflect the systemic redox status, because they are readily oxidized by reactive species [26]. Approximately 60% to 75% of circulating free thiols are present on the cysteine residues in albumin [27]. Additionally, free thiols are present in other low-molecular-weight proteins, such as cystatin C and glutathione, thereby also contributing to their role as antioxidants [28]. Levels of free thiols have been shown to be decreased in patients with active lupus nephritis [8], which was also shown as decreased total antioxidant capacity in patients with LN class III and IV [29] and in active SLE patients [30]. Next to this, natural thiol and total thiol levels were found to be significantly lower in SLE patients compared to patients with low disease activity and were regarded as ideal biomarkers of oxidative stress in SLE patients, which reflects disease activity and organ involvement [31]. In the present study, serum free thiol levels were also decreased in qSLE patients compared to HCs, whereas free thiol levels in iSLE patients were comparable to HCs, providing evidence that the antioxidant capacity in iSLE patients is not affected. It is known that patients with iSLE have fewer autoantibody specificities and lower incidence of photosensitivity, oral ulcers, and hematologic and immunologic disorders, as well as lower disease activity and lower ROS production, than SLE patients [31,32].

Reactive species and free radicals can interact with numerous molecules in SLE patients, creating deleterious cascade products that disrupt immune regulation and promote autoimmunity [6]. Elevated ROS production induced by H_2_O_2_ in peripheral blood lymphocytes from SLE patients has been demonstrated [33,34]. In line with previous findings, our study found that intracellular ROS levels in PBMCs were elevated in qSLE patients, whereas ROS levels in iSLE patients were comparable to HCs. We used frozen PBMCs for our studies for practical reasons, because the different laborious tests could not be performed on the same day of a patient visit. The PBMCs were thawed only once, and a study which investigated repeated freezing cycles showed a progressive increase in cell death percentages after three rounds of thawing, but the frequency of the main lymphocyte subsets was stable across the three thawing instances [35]. We acknowledge that results may be somewhat different between fresh and frozen cells, but PBMCs can be used when cells are thawed only once and checked for viability.

Many cellular antioxidant genes are regulated by NRF2 activation; therefore, it has a crucial role in maintaining cellular redox homeostasis [36]. Given its central role in redox regulation, activating the NRF2/KEAP1 pathway may help to resolve chronic inflammation, oxidative stress and tissue injury in SLE, potentially improving disease activity [21,37]. Notably, lupus-prone mouse studies showed that ROS levels were increased in cells with NRF2 deficiency; moreover, cells deficient in NRF2 were more sensitive to toxicity of various oxidants [21,38]. Consistent with increased ROS levels, mRNA expression of KEAP1 and CAT was decreased in SLE patients compared to HCs, whereas NRF2, HMOX1, and NQO1 expression showed a non-significant trend towards lower expression. Since NRF2 activity is primarily regulated through KEAP1-mediated control of NRF2 protein stability, unchanged NRF2 mRNA levels do not exclude alterations in NRF2 pathway activity. Although we observed reduced KEAP1 mRNA expression, the functional consequences at the protein level remain unclear. Reduced KEAP1 protein levels could potentially lead to decreased NRF2 degradation and NRF2 accumulation; however, this requires confirmation by protein-level analyses.

There was no differences between iSLE patients with or without HCQ use regarding free thiols, ROS levels, and NRF2 mRNA expression, although effects of HCQ on the mitochondrial antioxidant system in activated T cells have been reported [39]. In this study, however, clinically relevant concentrations of HCQ were added in vitro to cells, which might be different compared to ex vivo-treated cells. Moreover, patient numbers in our study were low.

DMF activates the NRF2 pathway while suppressing the NF-κB pathway and has been described to inhibit the secretion of proinflammatory cytokines by T cells [40]. Case reports have demonstrated that patients with cutaneous manifestations of SLE were successfully treated with DMF [41,42]. Although SFN has not been applied as an additional treatment in clinical trials, studies demonstrated that it has therapeutic effects on lupus-prone mouse models by reducing inflammation and oxidative stress [43,44,45]. HMOX1 has antioxidant effects by catalyzing degradation of heme into biliverdin, which is rapidly reduced to bilirubin by the enzyme biliverdin reductase [46]. It has been shown that in SLE patients, HMOX1 levels in monocytes were lower compared to HCs [47]. NRF2 activation boosted expression of HMXO1 and NQO1 in macrophages and PBMCs of patients with SLE in vitro [48], which is in line with our results in PBMCs and HaCaT cells. SNF seemed to be more potent than DMF in our experiments; however, these differences were not significant. Also, in other studies comparing both activators of NRF2, no clear conclusion can be drawn, as some studies showed more effects of SFN, and others showed equal effects [49,50,51]. Of note, CAT expression was downregulated in patients’ whole blood as well as in PBMCs treated with NRF2 activators. This may be due to polymorphisms in the CAT gene that are susceptible to oxidative stress, indicating that other mechanisms or factors may be involved in regulation of expression of the CAT gene in SLE [52,53,54].

We used treated HaCaT cells with NRF2 activators to investigate whether these skin cells could upregulate antioxidant genes, which was indeed the case. Next we looked at protein levels for NRF2 and HMOX1 in unaffected non-sun-exposed skin of patients and controls, between which we did not see differences; possibly, there were no lesions present in the skin. We could not find human studies exploring expression of protein NRF2 in skin, only mouse or in vitro studies.

Protein levels of NRF2 may be interesting considering the increasing interest in discovering novel NRF2 activators. Until now, most efforts have been devoted to the development of electrophilic drugs such as SFN and DMF that can target KEAP1 through covalent modifications on cysteine residues. However, off-target effects of these drugs prompted the development of an innovative strategy, the search for KEAP1-NRF2 protein–protein interaction (PPI) inhibitors [55]. These innovative activators might target NRF2 in a more selective way, leading to potentially improved drugs for application to a variety of diseases including SLE. Limitations of this study are the relatively low number of patients, and that no SLE patients with active disease were included, because in active disease the redox balance is even more disturbed. Another limitation is that the immunologic-specific role of NRF2 and the therapeutic effect of SFN and DMF were not elucidated in animal studies, and thus the significance of NRF2 and its agonists was only demonstrated in in vitro cellular models and PBMCs.

## 5. Conclusions

Oxidative stress is elevated, while antioxidative capacity is decreased, in qSLE patients compared to HCs, as shown by reduced thiol levels and by reduced expression of NRF2-related antioxidant genes such as KEAP1 and CAT. A reduced but not significant antioxidant capacity was seen in iSLE patients, while there was an inverse correlation with ROS levels. Both NRF2 activators, SFN and DMF, demonstrate the ability to upregulate expression of antioxidant genes, for instance HMOX1 and NQO1, in both PBMCs and HaCaT cells, highlighting their potential role to modulate oxidative stress pathways in SLE, which might also be beneficial even in the earliest stages of the disease.

## Figures and Tables

**Table 1:** Characteristics of subjects included for measurements of serum free thiols and whole blood oxidative stress genes.

	HCs (*n* = 21)	iSLE (*n* = 29)	qSLE (*n* = 28)	*p*-Value
Gender, female	17 (81%)	26 (90%)	22 (79%)	0.297
Age, years	46 (28–60)	44 (29–60)	41 (30–53)	0.936
BMI, kg/m^2^	22 ± 2.1	25 ± 4.9	25 ± 4.9	0.929
Disease duration, years	NA	1.2 (0.4–2.9)	3.1 (1.8–4.7)	0.003
Systolic blood pressure, mmHg	120 (110–124)	115 (110–133)	120 (110–130)	0.955
Diastolic blood pressure, mmHg	75 (69–81)	75 (70–80)	75 (62–80)	0.658
Current smoking, *n* (%)	0 (0%)	11 (38%)	4 (14%)	0.070
SLEDAI, score	NA	1 (0–2)	2 (2–4)	0.064
Skin involvement, positivity *n* (%)	NA	8 (28%)	3 (11%)	0.179
*Laboratory measurements*				
Serum creatinine, µmol/L	NA	69 (60–79)	77 (68–83)	0.041
Anti-dsDNA, IU/mL	NA	2 (1–5)	11 (3–33)	0.001
C3, g/L	NA	1.06 (0.98–1.19)	1.07 (0.84–1.16)	0.467
C4, g/L	NA	0.19 (0.15–0.24)	0.16 (0.13–0.21)	0.100
*Medication use*, *n* (%)				
NSAID	NA	7 (24%)	8 (29%)	0.468
Hydroxychloroquine	NA	9 (31%)	26 (93%)	<0.001
Prednisolone	NA	0 (0%)	7 (25%)	0.004
Methotrexate	NA	0 (0%)	2 (7%)	0.237
Mycophenolate mofetil	NA	0 (0%)	7 (25%)	0.004
Azathioprine	NA	0 (0%)	2 (7%)	0.237

Parameters are presented as mean ± SD, median (IQR) or number (%) of patients with iSLE and qSLE; NA: not applicable. Abbreviations: HCs: healthy controls; qSLE: quiescent systemic lupus erythematosus; iSLE: incomplete SLE; NA: not applicable; BMI, body mass index; SLEDAI, SLE disease activity index; Anti-dsDNA, anti-double stranded DNA; C3 and 4: complement 3 and 4.

**Table 2:** Characteristics of subjects included for the measurements of ROS levels in PBMCs and NRF2 activation experiments.

	HCs (*n* = 10)	iSLE (*n* = 20)	qSLE (*n* = 15)	*p*-Value
Gender, female	9 (90%)	18 (90%)	12 (80%)	0.102
Age, years	43 ± 12.7	44 ± 17.0	41 ± 17.1	0.536
BMI, kg/m^2^	23 ± 3.8	27 ± 5.8	25 ± 4.8	0.472
Disease duration, years	NA	1.9 (0.6–3.4)	3.4 (1.4–8.3)	0.053
Systolic blood pressure, mmHg	120 (113–131)	120 (111–138)	120 (110–140)	0.650
Diastolic blood pressure, mmHg	75 (70–85)	75 (70–80)	80 (75–85)	0.206
Current smoking, *n* (%)	0 (0%)	6 (30%)	5 (33%)	0.999
SLEDAI, score	NA	2 (0–2)	2 (0–4)	0.140
Skin involvement, positivity *n* (%)	NA	6 (30%)	1 (7%)	0.199
*Laboratory measurements*				
Serum creatinine, µmol/L	NA	64 (58–72)	67 (62–79)	0.133
Anti-dsDNA, IU/mL	NA	1 (1–4)	4 (1–23)	0.075
C3, g/L	NA	1.14 (1.01–1.38)	1.04 (0.90–1.20)	0.182
C4, g/L	NA	0.22 (0.18–0.29)	0.17 (0.11–0.24)	0.055
*Medication use*, *n* (%)				
NSAID	NA	5 (25%)	5 (33%)	0.712
Hydroxychloroquine	NA	5 (25%)	12 (80%)	0.002
Prednisolone	NA	0 (0%)	3 (20%)	0.070
Methotrexate	NA	0 (0%)	0 (0%)	NA
Mycophenolate mofetil	NA	0 (0%)	6 (40%)	0.003
Azathioprine	NA	0 (0%)	0 (0%)	NA

Parameters are presented as mean ± SD, median (IQR) or number (%) of patients with iSLE and SLE; Abbreviations: ROS: reactive oxygen species; PBMCs: peripheral blood mononuclear cells; HCs:, healthy controls; qSLE: quiescent systemic lupus erythematosus; iSLE: incomplete SLE; NA: not applicable; BMI: body mass index; SLEDAI: SLE disease activity index; Anti-dsDNA: anti-double stranded DNA; C3 and 4: complement 3 and 4.

## Data Availability

The data supporting the findings of this study are not publicly available due to technical limitations. However, the data are available from the corresponding author upon reasonable request.

## Supplementary Materials

The following supporting information can be downloaded at https://www.mdpi.com/article/10.3390/antiox15080995/s1, Figure S1: (A) Correlations between NRF2 and its related downstream genes; (B) Correlations between Nrf2 and its related downstream genes in quiescent SLE.
