# Supplementary material for: Oxidative Stress and NRF2-Mediated Redox Regulation in Incomplete Systemic Lupus Erythematosus and Systemic Lupus Erythematosus
Source: Antioxidants (Basel). 2026 Aug 11;15(8):995. doi: 10.3390/antiox15080995 (PMC13509375; doi:10.3390/antiox15080995)
Supplement: Supplementary file 1 [file antioxidants-15-00995-s001.zip › antioxidants-4448799-supplementary.pdf]

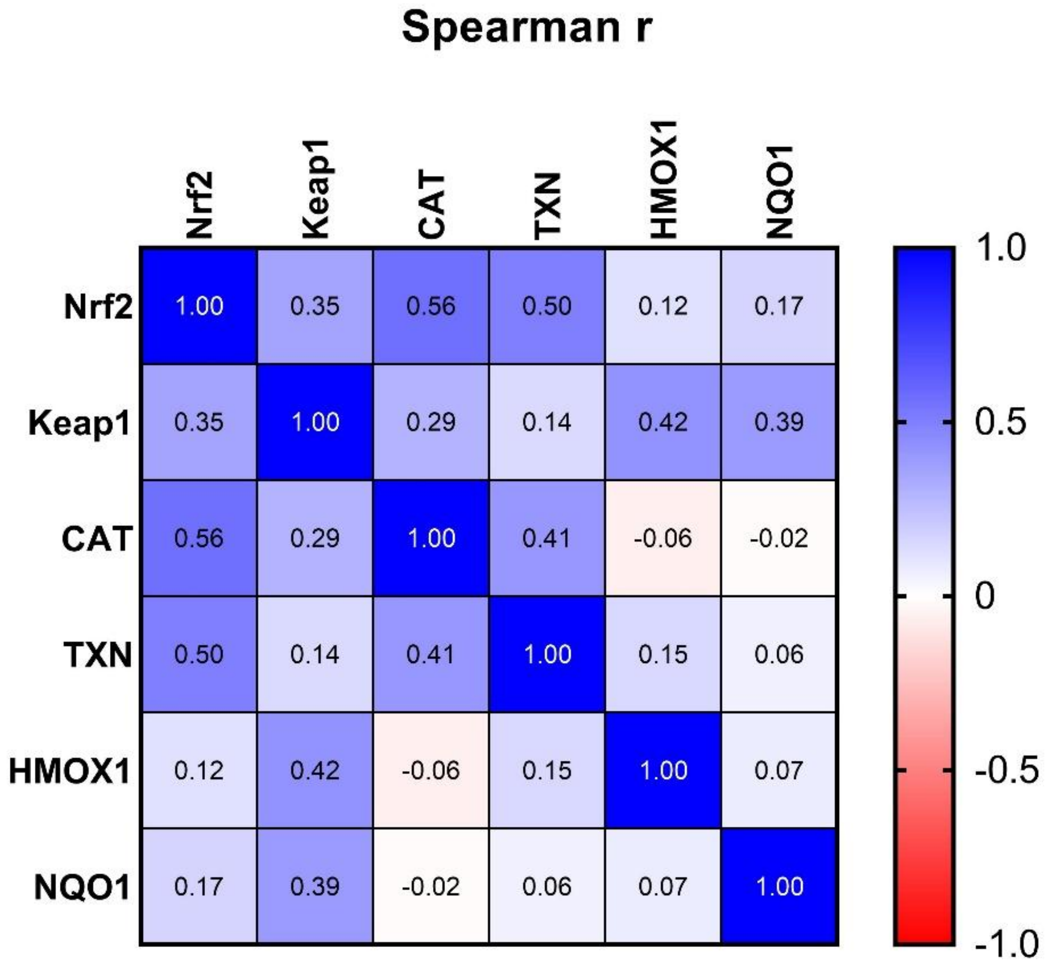

Figure S1A. Correlations between NRF2 and its related downstream genes in incomplete SLE

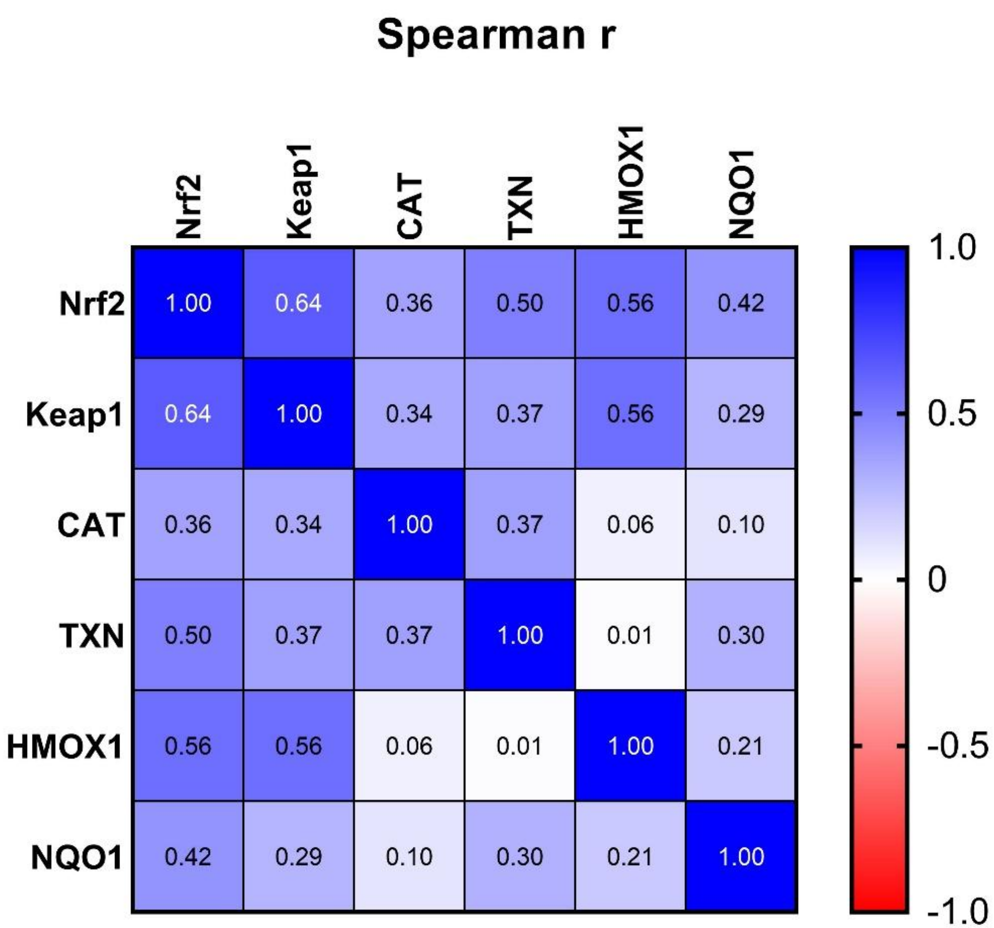

Figure S1B. Correlations between Nrf2 and its related downstream genes in quiescent SLE
